# Mating system and extra-pair paternity in the Fan-tailed Gerygone *Gerygone flavolateralis* in relation to parasitism by the Shining Bronze-cuckoo *Chalcites lucidus*

**DOI:** 10.1371/journal.pone.0194059

**Published:** 2018-03-08

**Authors:** Katarzyna Bojarska, Ralph Kuehn, Małgorzata A. Gazda, Nozomu J. Sato, Yuji Okahisa, Keita D. Tanaka, Alfredo Attisano, Roman Gula, Keisuke Ueda, Jörn Theuerkauf

**Affiliations:** 1 Museum and Institute of Zoology, Polish Academy of Sciences, Warsaw, Poland; 2 Unit of Molecular Zoology, Chair of Zoology, Department of Animal Science, Technische Universität München, Freising, Germany; 3 Department of Fish, Wildlife & Conservation Ecology and Molecular Biology Program, New Mexico State University, Las Cruces, New Mexico, United States of America; 4 Department of Life Sciences, Rikkyo University, Tokyo, Japan; Hungarian Academy of Sciences, HUNGARY

## Abstract

Extra-pair copulation can increase genetic diversity and offspring fitness. However, it may also increase intra-nest variability in avian hosts of brood parasites, which can decrease the discrimination ability of host parents towards the parasite. In New Caledonia, the Fan-tailed Gerygone (*Gerygone flavolateralis*), which is parasitized by the Shining Bronze-cuckoo (*Chalcites lucidus*), has two nestling morphs, dark and bright, that can occur in monomorphic and polymorphic broods. Gerygone parents recognize and eject parasite nestlings from their nest, but the presence of polymorphic broods may increase the chances of recognition errors. Using 17 microsatellite markers, we investigated the mating system of the Fan-tailed Gerygone to understand the mechanisms underlying nestling polymorphism. We hypothesised that extra-pair copulations would lead to a higher proportion of polymorphic broods caused by higher genetic variability, thus creating a trade-off between genetic benefits and host defence reliability. Extra-pair paternity occurred in 6 of 36 broods, which resulted in 6 of 69 offspring sired by extra-pair males. Broods with and without mixed paternity were comparably often parasitized. Extra-pair paternity did not influence the proportions of bright, dark and polymorphic broods. Compared to bright siblings in polymorphic broods, dark nestlings tended to have lower heterozygosity, particularly in loci associated with skin coloration. The results also suggested that there is no obstacle for genetic exchange between individuals from forest and savannah, possibly due to dispersal of offspring. We conclude that the Fan-tailed Gerygone is a socially monogamous species with a low rate of extra-pair paternity compared to closely related species. Extra-pair paternity increased offspring genetic variability without measurable associated costs by brood parasitism. The results highlight the importance of studying host mating systems to assess the trade-offs between host defence and offspring fitness in co-evolutionary arms races.

## Introduction

Avian brood parasites lay eggs in the nests of other birds and leave the young to the care of their hosts [1]. The fitness cost of brood parasitism can be high, thus hosts are under selection to evolve defences against parasitism [2]. These defences can act at any moment of the breeding cycle, including frontline defences during the period before egg laying [3–5], during the egg stage [6–9] or during the nestling stage [10–14]. Cooperatively breeding species are more often hosts to brood parasites than non-cooperative species, possibly because more individuals care better for the parasite [15]. On the other hand, some cooperative breeders and colonial nesters more effectively defend their nests against parasites as more individuals are involved in the surveillance [16,17]. In monogamous species, male and female of a host pair may have different roles in nest guarding against brood parasitism [18]. If this involves increased nest attendance by males, this may reduce the time males can guard females to impede extra-pair copulation [19]. Non-cooperative polygynous species that are parasitized have a similar problem as their mating strategy decreases the male investment in nest defence [20], which can result in increased parasitism rates [21]. Despite its importance, there is still little research devoted to mating systems of hosts in Oceania, where the arms race between cuckoos and their hosts has escalated to the nestling stage [10,13] and cooperative breeding is wide-spread [22].

The Fan-tailed Gerygone *Gerygone flavolateralis* from New Caledonia, which is exclusively parasitized by the Shining Bronze-cuckoo *Chalcites lucidus*, is an egg acceptor as the Grey Gerygone *Gerygone igata* from New Zealand [23]. However, in contrast to the Grey Gerygone, the Fan-tailed Gerygone ejects parasite nestlings [14] as do two other Australian gerygone species [12,13]. Nestlings of bronze-cuckoos in Australia therefore mimic host nestlings [24] and this also occurs in New Caledonia [14]. Nestlings of Fan-tailed Gerygone, however, occur in two distinct skin colour phenotypes, dark (dark grey) and bright (pinkish grey), in monomorphic and polymorphic broods [14]. Despite cuckoo mimicry and polymorphic broods, Fan-tailed Gerygone parents recognize and eject the parasite nestlings from the nest in any type of brood [Attisano et al. unpublished data]. Nestling polymorphism is a rare occurrence in birds [25], thus the strong selective pressure by parasitism may explain the presence of two host nestling morphs as an evolved host defence [14]. However, the presence of two nestling morphs in the same brood may increase the chances of recognition errors [14] and thus constrain the advantage of host chick polymorphism. The inheritance mechanisms of nestling skin colour in Fan-tailed Gerygones are unknown, but if skin colour is not inherited solely by the female, then extra-pair paternity could lead to different skin colour of chicks within a brood or within broods of the same pair.

The application of molecular tools in studying breeding in birds allows distinguishing between social and genetic monogamy [26] or recognising the relatedness in cooperative breeding groups [27]. Molecular analyses revealed that even among socially monogamous bird species, genetic monogamy occurs in less than 25% of cases [28]. Females involve in extra-pair copulations because they can gain indirect benefits, such as offspring heterozygosity and other fitness benefits, parasite resistance, and genetic pathogen resistance, but on the other hand, females should avoid disadvantageous consequences of extra-pair copulations, such as abandonment by their social mate [29–31]. In this study, we used microsatellite markers to assess the mating system of the Fan-tailed Gerygone and to understand the genetic mechanisms underlying nestling polymorphism. Our primary objectives were to investigate (1) if the Fan-tailed Gerygone is socially or genetically monogamous, (2) if extra-pair copulations affect rates of offspring skin colour polymorphism, and (3) if extra-pair offspring have higher genetic variability than other offspring. We hypothesised that extra-pair copulations would lead to higher genetic variability but also to a higher proportion of mixed broods. Females would then benefit from extra-pair mating, but this might be counter-balanced by the costs of recognition errors in polymorphic broods.

## Methods

We conducted fieldwork on the main island (Grande Terre) of New Caledonia during six breeding seasons (September-January) from 2011/12 to 2017/18. We collected samples in the Parc des Grandes Fougères (PGF) and surroundings (21°37–40’S, 165°45–47’E). The southern province of New Caledonia issued all permits for field work (3045–2011, 2437–2012, 2532–2013, 2801–2014, 2476–2015, 2372–2017). Handling and collection of the nestlings was performed in accordance with the guidelines and regulations outlined in the permits. The 1^st^ Warsaw Local Ethics Committee for Animal Experimentation approved all procedures. The PGF is a protected area of mainly rainforest, with elevations ranging from 400 to 600 m. The surrounding area is located at lower altitudes (200–400 m) and consists of savannah with small patches of secondary forest and open grassland.

We searched for active Fan-tailed Gerygone nests by following adult birds returning to their nests and then checked for the presence of Shining Bronze-cuckoo eggs. We captured host parents returning to their nest by placing mist nets within 1–2 meters from the nest entrance, and colour-banded adults and nestlings to allow individual recognition. We collected blood samples (10–30 μl) with capillaries from all nestlings (8–10 days old) and from adult individuals caught at the nest and stored them in ethanol or RNA later as described in Gazda et al. [32]. In addition, we collected tissue samples of 31 nestlings that we found dead of unknown reasons in the nest. We took genetic samples of 282 individuals (101 adults and 181 offspring) from 99 breeding attempts. Of these, we used for further analyses 127 individuals of 29 pairs and their offspring (36 breeding attempts, 69 nestlings, 1–3 per brood, mean = 1.9, SD = 0.7), for which both mother and father were genetically confirmed. As the skin of Fan-tailed Gerygone nestlings has a discrete colour variation at hatching [14], we categorized them to either the bright or the dark morph by visual inspection. We were able to recognize skin coloration in 64 of 69 genotyped offspring.

We genotyped 17 microsatellite markers of all sampled individuals, following the methods described in Gazda et al. [32]. Then, we determined paternity within each breeding attempt through a likelihood-based approach using CERVUS 3.0.7 [33]. We checked for all nestlings if the female captured near an active nest was in fact the mother; in all cases, the parentage analysis confirmed maternity. We considered a male as the father of a chick if CERVUS assigned him as its most likely father and the pair LOD score (log-likelihood ratio for a parent-offspring relationship) was within the 95% confidence interval.

We considered a brood as mixed-paternity when the male caught at the nest was the most likely father of only some of the offspring based on the results of the paternity analysis. For the analyses of genetic parameters, we used the 36 breeding attempts (127 individuals) for which both mother and father were genetically confirmed. These 36 breeding attempts were from 24 pairs followed during a single breeding attempt, three pairs during two breeding attempts and of two pairs during three breeding attempts.

We calculated mean allelic richness, expected and unbiased observed heterozygosity for each locus and each breeding attempt with FSTAT 2.9.3.2 [34,35]. We estimated the relatedness (F value) among individuals within each breeding attempt using 2MOD software [36], which refers to the probability that two genes share a common ancestor within a population. To investigate the genetic structure between and within the two main study areas (forest and savannah), and among and within gerygone breeding attempts, we carried out an analysis of molecular variance (AMOVA) with ARLEQUIN 3.1 [37]. To visualize the genetic structure among and within breeding attempts, we performed a discriminant analysis of principal coordinates [38] on Euclidean distances between individuals using the ADEGENET 2.0.1 package [39,40] in R 3.3.2 [41]. We transferred the first three principal coordinates into colours using a Red-Green-Blue (RGB) algorithm with ADEGENET and ade4 [39,42]. We calculated individual estimates of genetic diversity: internal relatedness (a multilocus heterozygosity measure developed by Amos et al. [43]) and standardized heterozygosity using the R package Rhh 1.0.2 [44]. To assess the association between genotype and chick skin coloration, we used CLUMP 2.4 software, which compares allele frequencies with χ^2^ tests [45].

We compared the breeding attempt-based genetic diversity indices (allelic richness, expected heterozygosity, observed heterozygosity, relatedness between individuals within each breeding attempt) between breeding attempts with extra-pair and without extra-pair young. We additionally compared individual heterozygosity indices (internal relatedness and standardized heterozygosity) between the nestlings sired by their social father and by extra-pair fathers, and between nestlings of dark and bright skin. For these comparisons, we used genetic diversity indices based on all microsatellites and based on loci classified by CLUMP as associated to nestling skin colour.

## Results

### Spatial population structure

**Fig 1:**
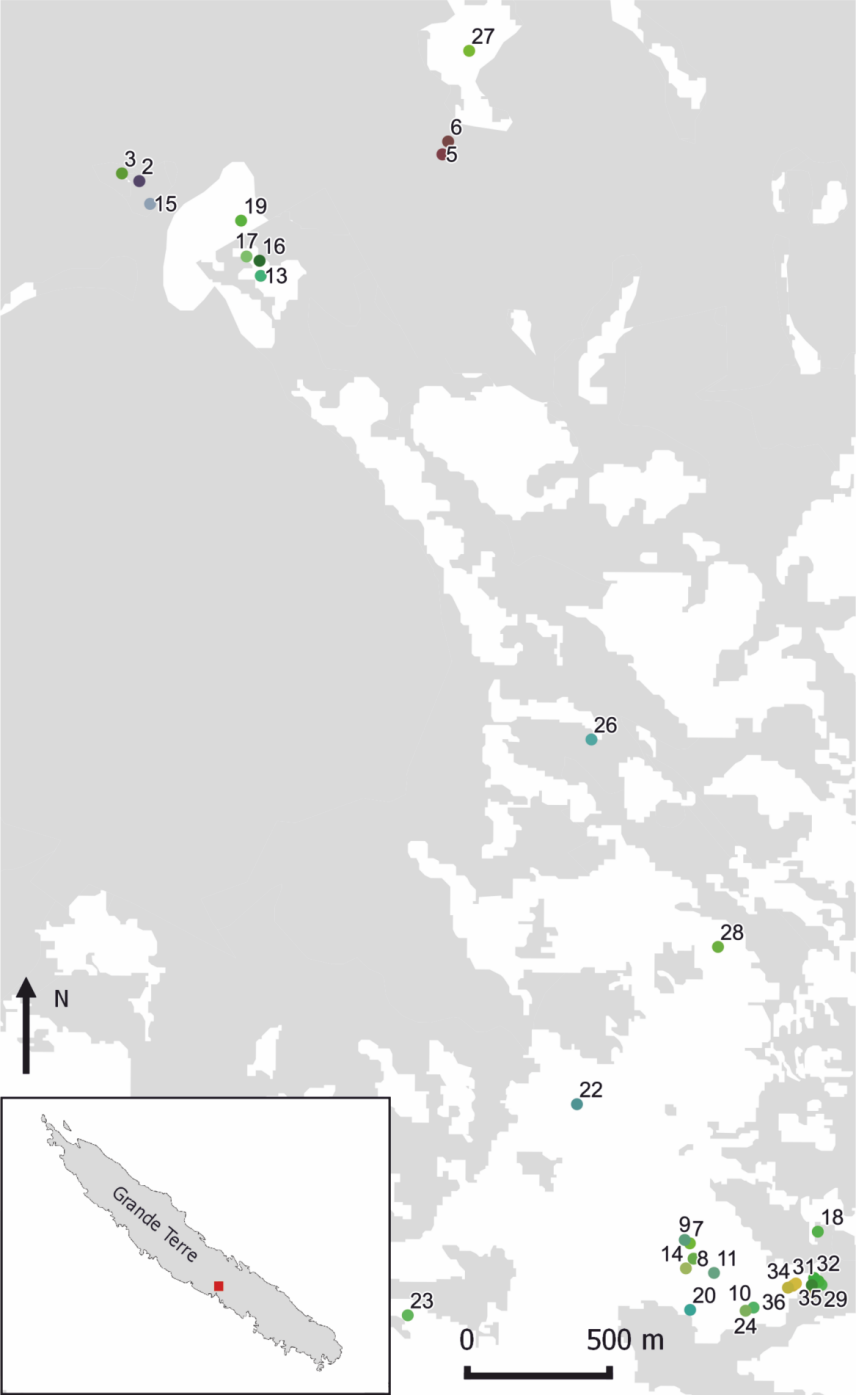
Locations of sampled Fan-tailed Gerygone nests in the Parc des Grandes Fougères and surroundings. The colours of dots indicate the mean genetic constitution of breeding attempts, based on Principal Coordinates calculated on Euclidean distances and transformed into RGB values. White background indicates open areas, grey forest.

Territories of breeding pairs were often adjacent and the distance between active nests was in some cases as close as 20 m. Breeding pairs remained together and showed a strong site fidelity over breeding seasons: nests of pairs that we followed for multiple breeding attempts were within 50 m (mean = 24 m, SD = 15 m). Offspring dispersed far from their parental territories. In only one case, we found a female offspring four years after dispersal. She was nesting in savannah at a straight-line distance of 4.1 km from her natal nest, which was in forest. We found no spatial differentiation in population structure between the two main study areas (forest and savannah) based on the hierarchical analysis of molecular variances among and within breeding attempts and between and within areas (Fig 1). AMOVA revealed that 97% of all genetic variance occurred among individuals, 20% among breeding attempts within each area and only 0.4% between the two study areas, minus 18% due to differences among individuals within breeding attempts.

### Brood paternity

**Fig 2:**
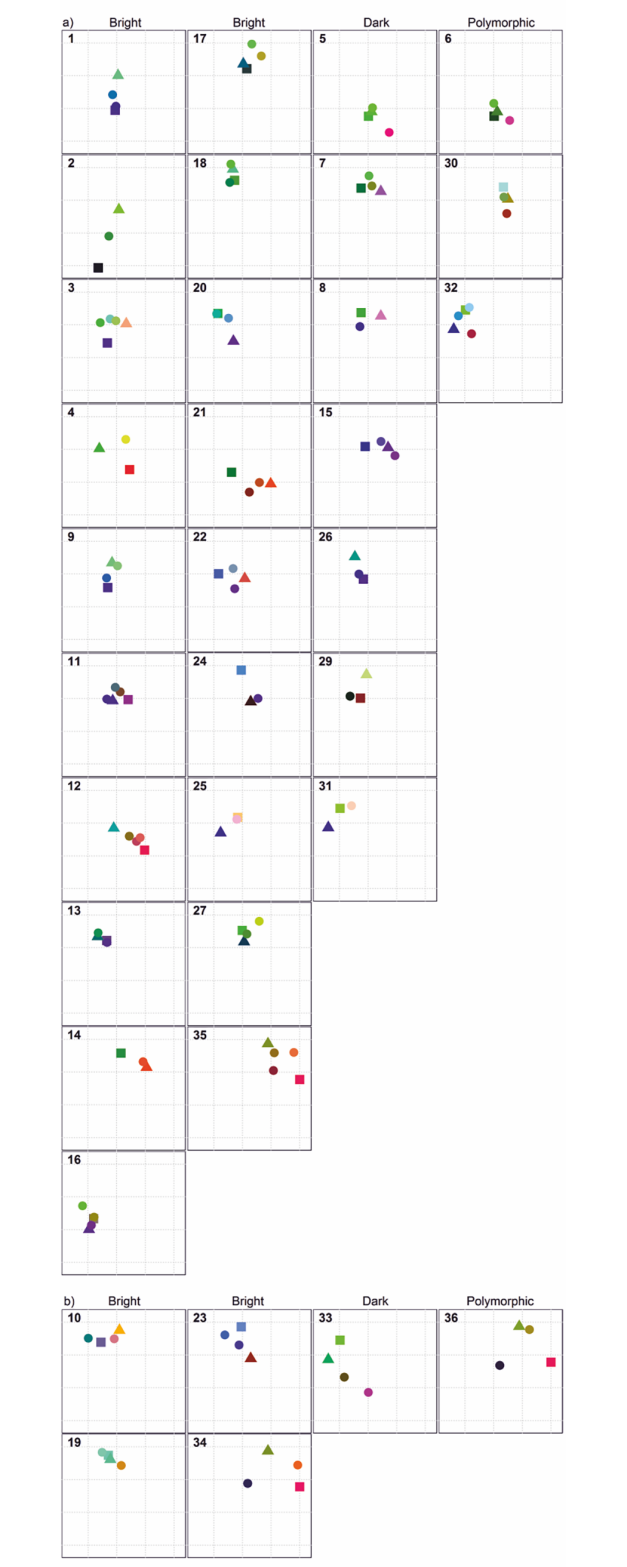
**Genetic constitution of 127 Fan-tailed Gerygones of 36 breeding attempts in New Caledonia with single (a) and mixed (b) paternity.** First two columns consist of broods with bright skin coloration, third column–dark skin coloration, and fourth column–polymorphic broods. The number in upper left corner of each plot corresponds to breeding attempt ID in Fig 1 and Table 1. Squares indicate females, triangles males, and circles offspring. Position of the individuals within the graph is determined by two first Principal Coordinates calculated based on Euclidean distances. The colours correspond to the three first Principal Coordinates transformed into RGB.

Extra-pair offspring occurred in 17% (6 of 36) of breeding attempts and in 17% (5 of 29) of breeding pairs (Table 1, Fig 2). Nine percent (6 of 69) of all offspring were extra-pair young. All sampled mixed-paternity broods consisted of two nestlings, thus contained one extra-pair and one within-pair young. Three of five pairs that we monitored during multiple breeding attempts had mixed-paternity broods: once in a pair monitored for two years (breeding attempts 23 and 24 in Fig 1, Fig 2 and Table 1), and once (breeding attempts 31–33) and twice (breeding attempts 34–36) in two pairs monitored for three years. All five pairs monitored for more than one breeding season were socially monogamous throughout the study period. As only one of these females had extra-pair offspring in multiple broods, we could not quantitatively assess if extra-pair young were sired by the same or different extra-pair males. However, as we never observed more than two adult individuals at nests, there is no indication for cooperative breeding in the Fan-tailed Gerygone.

**Table 1:** Individual values and averages (with 95% confidence intervals) for 36 breeding attempts of 29 Fan-tailed Gerygone pairs in New Caledonia, during the breeding seasons from 2011/12 to 2015/16, based on 17 microsatellite markers. ID: number of breeding attempt (corresponding to Fig 1); Pair: number of breeding pair; N: number of sampled individuals in the breeding attempt (including the breeding pair); Allele number: mean number of alleles per locus; Allelic richness: mean allelic richness per locus; HE: unbiased expected heterozygosity; HO: observed heterozygosity, F value: relatedness between individuals; Paternity: all offspring sired by one male (within-pair: WP) or two males (extra-pair: EP); offspring skin colour (poly means both colours occur in a brood); Parasitism: whether a brood was parasitized by Shining Bronze-cuckoo (1 –parasitized, 0 –not parasitized); Genetic constitution: colour based on the first three Principal Coordinates calculated on Euclidean distances and transformed into RGB values.

ID	Pair	N	Allele number	Allelic richness	HE	HO	F value	Paternity	Skin colour	Parasitism	Genetic constitution		
1	1	4	2.53	1.54	0.54	0.65	0.146	WP	bright	0			
2	2	3	2.35	1.53	0.53	0.51	0.150	WP	bright	0			
3	3	5	2.82	1.56	0.56	0.66	0.102	WP	bright	0			
4	4	3	2.29	1.58	0.58	0.71	0.199	WP	bright	0			
5	5	4	2.71	1.71	0.59	0.71	0.139	WP	dark	0			
6	5	4	2.82	1.49	0.60	0.71	0.165	WP	poly	1			
7	6	4	2.82	1.65	0.59	0.74	0.149	WP	dark	0			
8	6	3	2.82	1.64	0.66	0.78	0.055	WP	dark	1			
9	7	4	3.18	1.59	0.68	0.81	0.108	WP	bright	0			
11	9	5	3.29	1.59	0.62	0.73	0.130	WP	bright	0			
12	10	5	2.47	1.72	0.48	0.60	0.236	WP	bright	0			
13	11	4	2.82	1.68	0.59	0.69	0.156	WP	bright	0			
14	12	3	2.65	1.62	0.60	0.65	0.109	WP	bright	0			
15	13	4	2.65	1.58	0.56	0.71	0.307	WP	dark	0			
16	14	5	2.29	1.62	0.49	0.64	0.317	WP	bright	0			
17	15	4	3.12	1.48	0.61	0.71	0.159	WP	bright	0			
18	16	4	2.59	1.59	0.51	0.57	0.181	WP	bright	0			
20	18	4	2.35	1.56	0.50	0.64	0.299	WP	bright	0			
21	19	4	2.59	1.49	0.52	0.65	0.181	WP	bright	0			
22	20	4	3.00	1.61	0.65	0.75	0.118	WP	bright	0			
24	21	3	3.24	1.56	0.71	0.78	0.124	WP	bright	1			
25	22	3	2.88	1.50	0.65	0.78	0.268	WP	bright	1			
26	23	3	2.88	1.53	0.64	0.75	0.062	WP	dark	0			
27	24	4	2.94	1.52	0.58	0.69	0.164	WP	bright	0			
28	25	4	2.82	1.70	0.53	0.59	0.099	WP	unknown	0			
29	26	3	2.29	1.56	0.56	0.71	0.099	WP	dark	0			
30	27	4	2.65	1.61	0.55	0.66	0.115	WP	poly	0			
31	28	3	2.47	1.55	0.53	0.63	0.059	WP	dark	1			
32	28	5	2.65	1.66	0.49	0.61	0.180	WP	mixed	0			
35	29	5	2.94	1.62	0.61	0.78	0.156	WP	bright	0			
**Average WP ±95%CI**			**2.73±0.10**	**1.59±0.02**	**0.58±0.02**	**0.69±0.03**	**0.158±0.025**			**0.17**			
10	8	4	3.06	1.60	0.62	0.71	0.092	EP	bright	0			
19	17	4	2.94	1.60	0.56	0.63	0.198	EP	bright	0			
23	21	4	3.41	1.51	0.62	0.69	0.122	EP	bright	0			
33	28	4	3.06	1.53	0.61	0.66	0.174	EP	dark	0			
34	29	4	3.53	1.65	0.66	0.74	0.043	EP	bright	1			
36	29	4	3.59	1.61	0.70	0.79	0.039	EP	poly	0			
**Average EP ±95%CI**			**3.26±0.22**	**1.58±0.04**	**0.63±0.04**	**0.70±0.05**	**0.111±0.053**			**0.17**			

**Fig 3:**
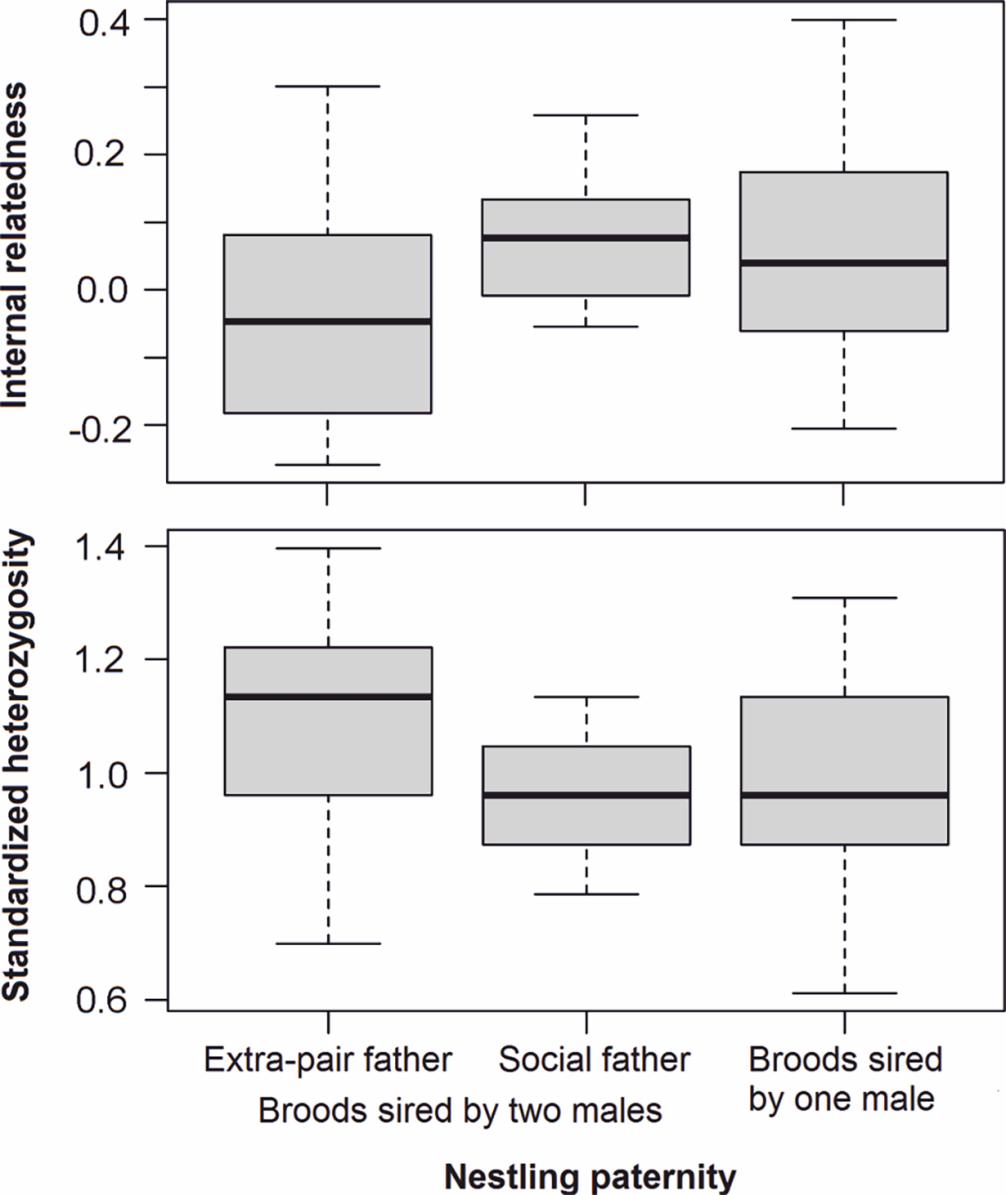
Internal relatedness and standardized heterozygosity in Fan-tailed Gerygone nestlings from six mixed-paternity broods (offspring sired either by an extra-pair father (n = 6) or by their social father (n = 6)) and in single-paternity broods (n = 57) based on the analysis of 17 microsatellite loci.

Mixed-paternity broods had higher inter-individual variation in genetic constitution (Fig 2) and higher mean number of alleles per locus (Table 1) than other broods. Allelic richness, expected and observed heterozygosity, and F values were not different in breeding attempts with mixed and single paternity (Table 1). Fan-tailed Gerygone clutches with mixed paternity (1 of 6) were as often parasitized as single-paternity clutches (5 of 30). The extra-pair young tended to have lower values of internal relatedness and higher values of standardized heterozygosity than within-pair offspring (Fig 3), although the difference was not significant (t-test, internal relatedness: P = 0.196; standardized heterozygosity: P = 0.176). In pairwise comparison of extra-pair offspring and their half-siblings, internal relatedness (paired t-test, P = 0.398) and standardized heterozygosity (P = 0.320) were not different.

### Skin coloration

**Fig 4:**
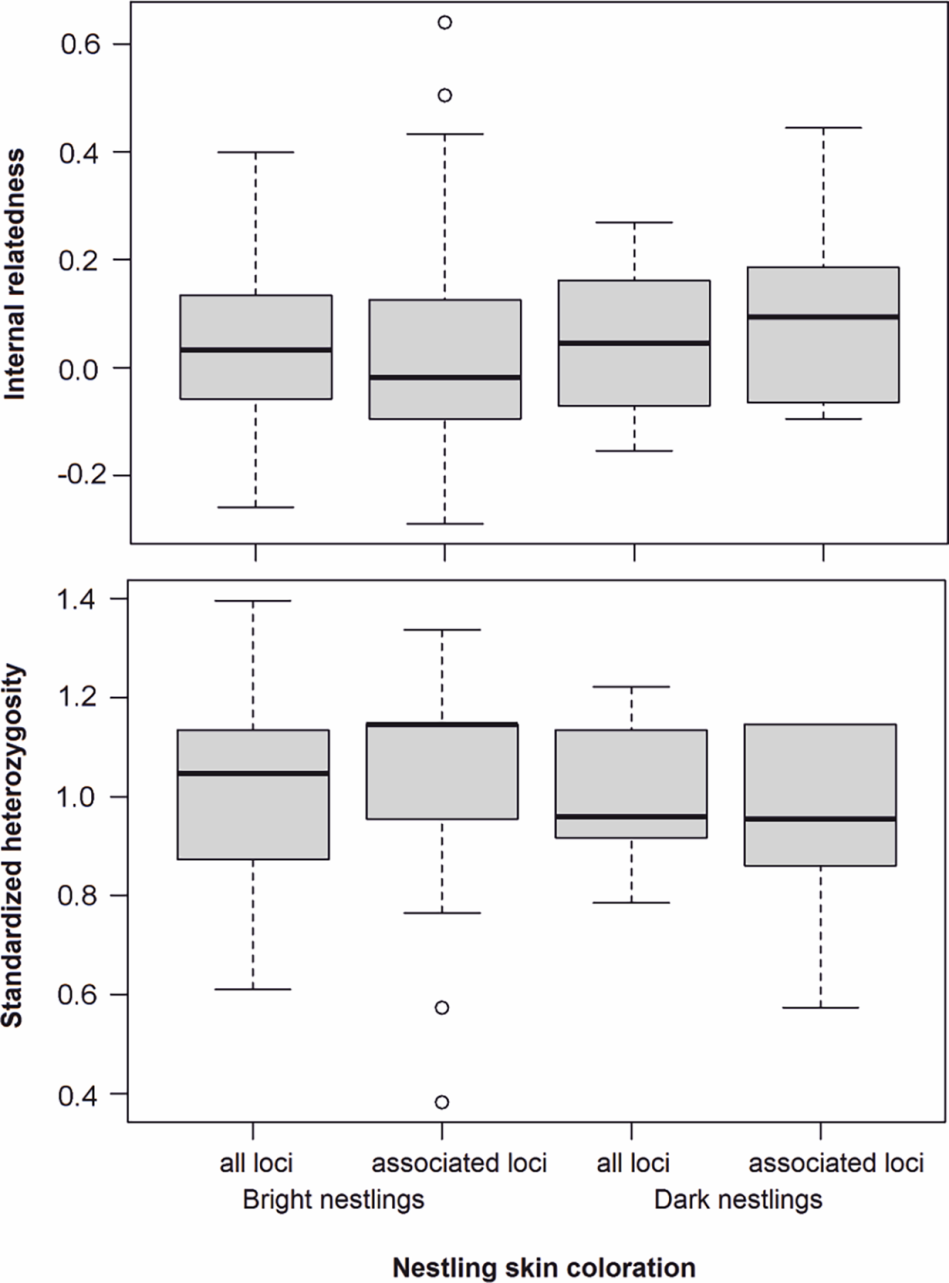
Internal relatedness and standardized heterozygosity in Fan-tailed Gerygone nestlings in New Caledonia grouped by skin coloration (49 bright and 15 dark), based on analysis of all 17 microsatellite loci and seven loci associated with skin colour.

Extra-pair paternity did not influence the proportions of bright, dark and polymorphic broods or nestlings (Fisher’s exact test, P = 0.228 for broods, P = 0.999 for nestlings, Table 2). Brood coloration even varied within broods of the same parents: two of three pairs with two breeding attempts with single paternity had each one dark and one polymorphic brood. The CLUMP analysis indicated that seven of 17 microsatellite loci were associated with chick skin coloration (Table 3). The individual heterozygosity indices (internal relatedness and standardised heterozygosity) based on all 17 loci were similar in the 49 bright and the 15 dark nestlings (Fig 4). Comparison of individual heterozygosity indices based only on the seven loci associated with skin colour showed slightly lower heterozygosity in dark chicks (Fig 4). Host parents never ejected their own chicks or abandoned their nest regardless of having polymorphic or monomorphic broods.

**Table 2:** Number (proportion) of bright, dark and polymorphic Fan-tailed Gerygone broods and number of bright and dark nestlings in single-paternity broods and mixed-paternity broods.

		Broods		Nestlings	
	bright	dark	polymorphic	bright	dark
Single paternity	19 (66%)	7 (24%)	3 (10%)	40 (76%)	13 (24%)
Mixed paternity	4 (66%)	1 (17%)	1 (17%)	9 (82%)	2 (18%)

**Table 3:** Genetic diversity measures of 17 microsatellite markers in 69 Fan-tailed Gerygone nestlings and the results of association analysis performed in CLUMP 2.4 with skin coloration of the offspring. HE: unbiased expected heterozygosity; HO: observed heterozygosity; p: level of significance of the chi-squared value from the table obtained by comparing χ^2^ of the allele frequencies in dark and bright chicks with χ^2^ calculated based on simulated (100x) tables with the same row and column totals, after collapsing low allele frequencies (<5%, [45]). Significant results (loci associated with skin coloration) after Bonferroni correction (adjusted P < 0.003) are marked with asterisk.

Marker	HO	HE	p
loc1	0.86	0.72	0.003
loc2	0.87	0.72	0.000*
loc3	0.77	0.63	0.002*
loc4	0.48	0.41	0.304
loc5	0.79	0.64	0.101
loc6	0.77	0.59	0.001*
loc7	0.85	0.65	0.003
loc8	0.52	0.42	0.002*
loc9	0.68	0.57	0.010
loc10	0.54	0.46	0.128
loc11	0.85	0.69	0.001*
loc12	0.72	0.60	0.006
loc13	0.80	0.65	0.000*
loc14	0.32	0.27	0.212
loc15	0.80	0.66	0.000*
loc16	0.24	0.20	0.017
loc17	0.84	0.74	0.009

## Discussion

Although Fan-tailed Gerygones of our study lived in different habitats, the lack of genetic differentiation between the forest and savannah areas suggests that there is no habitat barrier for genetic exchange. Adults were territorial during the breeding season and used the same nesting territory over multiple years. By contrast, the only banded offspring that we found after dispersal established a territory over 4 km away and in a different habitat than the territory of its parents. This indicates that most offspring probably disperse relatively far from their natal nests and potentially to different habitats. This explains why we did not find habitat-specific proportions of bright and dark chicks. It is therefore unlikely that habitat-specific adaptations against parasitism can become evolutionary stable on a local scale.

Several studies [29,46,47] found that extra-pair offspring had higher genetic variability and associated higher fitness than offspring sired by the social father. Also Fan-tailed Gerygone females seem to obtain genetic benefits from extra-pair paternity in terms of higher genetic variability, both at the breeding attempt (number of alleles per locus) and individual (offspring heterozygosity) level. However, the Fan-tailed Gerygone is a socially monogamous species with relatively low extra-pair paternity rates (9% of offspring; 17% of broods) compared to other passerine birds, a quarter of which have extra-pair paternity rates exceeding 25% of offspring (reviewed in Griffith et al. [28] and Lifjed et al. [48]). Only two Australian species of the Acanthizidae family have also been the subject of extra-pair paternity studies. The White-browed Scrubwren, *Sericornis frontalis*, employs either a cooperative breeding or monogamous breeding strategy, with extra-pair paternity rates in monogamous pairs at 24% of offspring and 42% of broods [49]. The extra-pair paternity rates found in our study were closer to those observed in the Brown Thornbill, *Acanthiza pusilla*, (6% of offspring; 12% of broods), a species with a developed female guarding behaviour [50]. Although we did not specifically study mate guarding in the Fan-tailed Gerygone, our field observations suggest that the pairs spend the majority of time in close proximity to each other. Therefore, mate guarding by males may explain the low extra-pair paternity rates that we found in Fan-tailed Gerygones despite close distances between nests of neighbouring breeding pairs, which have been reported to increase probability of extra-pair copulations in birds [51]. The ability of parents to distinguish their offspring from the parasite may be limited in a polymorphic brood, and the potential costs of misidentification may be higher than genetic benefits from extra-pair paternity. However, at the low mixed-paternity rates that we found, there was no effect of extra-pair paternity on the frequency of polymorphism in broods or offspring.

The proportion of microsatellite loci that were associated with skin coloration was surprisingly high. Lower levels of heterozygosity in loci associated with skin coloration observed in dark chicks suggest that color-coding genes are homozygotic in dark individuals, therefore dark skin colour may be a recessive trait, inherited in a recessive-dominant mode, as assumed by Sato et al. [14]. A study focused on the segregation of genes responsible for skin colouration in nestlings would provide insight into the inheritance mechanism of this trait and help to understand the potential consequences of extra-pair copulation for this host-parasite arms race.

We conclude that nestling polymorphism is unlikely to act as an evolutionary driver against extra-pair copulations in the Fan-tailed Gerygone, and extra-pair paternity rates are low due to factors other than brood parasitism. Regarding the chick coloration inheritance mechanisms, our findings based on genetic data are in concordance with the results of quantitative genetic results (frequency of skin colour) and simulations described in Sato et al. [14].

## Supporting information

S1 File Information on 141 samples of 127 Fan-tailed Gerygones (29 breeding pairs and their 69 offspring, 36 breeding attempts) in New Caledonia. ID: ID of individual, nest: nest number, breeding attempt: number of breeding attempt, status: parent or nestling, sex: F = female, M = male, habitat: type of habitat surrounding the nest, brood polymorphism: 0 = monomorphic, 1 = polymorphic), brood paternity: 0 = all chicks sired by one male, 1 = chicks sired by two males, offspring paternity: 0 –offspring sired by the social male, 1 –offspring sired by an extra-pair male, offspring skin colour: bright or dark, parasitism: 0 = breeding attempt not parasitized, 1 = parasitized, X and Y: coordinates in UTM WGS 84, zone 58S, date: sampling date, loc 1 –loc 17: values for 17 microsatellite markers, IR all loci: internal relatedness in nestlings based on all 17 microsatellite loci, IR colour-associated loci: internal relatedness in nestlings based on 7 loci associated with skin coloration, SH all loci: standardized heterozygosity in nestlings based on all 17 microsatellite loci, SH colour-associated loci: standardized heterozygosity in nestlings based on 7 loci associated with skin coloration.
(XLSX)
